# Interannual Variation in Seed Traits of *Cedrela* Species: Implications for Conservation in the Context of Climate Change

**DOI:** 10.3390/plants15030380

**Published:** 2026-01-26

**Authors:** Guadalupe Galíndez, Ana Álvarez, Diana Ceccato, Victoria Rivero, Gisela Malagrina, Tania Bertuzzi, Pablo Saravia, Stavros Nicolás Sola, Carol C. Baskin, Luis Fornes

**Affiliations:** 1 Facultad de Ciencias Naturales, Universidad Nacional de Salta, Av. Bolivia 5150, Salta 4400, Salta, Argentina; galindez@natura.unsa.edu.ar; 2 Centro Científico Tecnológico (CCT)-Salta-Jujuy, Consejo Nacional de Investigaciones Científicas y Técnicas (CONICET), Rivadavia 940, Salta 4400, Salta, Argentina; 3 Banco Base de Germoplasma, Instituto de Recursos Biológicos, Centro de Investigación de Recursos Naturales, Instituto Nacional de Tecnología Agropecuaria (IRB-CIRN-INTA), De los Reseros y N. Repetto s/n, Hurlingham 1686, Buenos Aires, Argentina; 4 Agencia de Extensión Rural San Julián, Estación Experimental Santa Cruz, Instituto Nacional de Tecnología Agropecuaria (AER-EEA-INTA Santa Cruz), Av. San Martín 1280, San Julián 9310, Santa Cruz, Argentina; 5 Facultad de Ciencias Agrarias, Universidad Nacional de Catamarca, Maestro Quiroga 50, Catamarca 4700, Catamarca, Argentina; 6 Estación Experimental Agropecuaria Famaillá, Instituto Nacional de Tecnología Agropecuaria (EEA-INTA Famaillá), Ruta 301–km 32, Famaillá 4132, Tucumán, Argentina; 7 Instituto de Clima y Agua, Centro de Investigación de Recursos Naturales, Instituto Nacional de Tecnología Agropecuaria (CIRN-INTA), De los Reseros y N. Repetto s/n, Hurlingham 1686, Buenos Aires, Argentina; 8 Department of Biology, University of Kentucky, Lexington, KY 40506-0225, USA; 9 Department of Plant and Soil Sciences, University of Kentucky, Lexington, KY 40546-0321, USA

**Keywords:** forest species, germination, seed ageing, seed bank, seed mass, viability equation, viability loss index

## Abstract

Climate change is altering temperature and precipitation regimes in Argentina, with potential consequences for regeneration and persistence of forest tree species, emphasizing the importance of ex situ seed conservation. We evaluated interannual variation in seed traits, desiccation tolerance, storage behavior, and longevity of *Cedrela balansae* C. DC. and *C. fissilis* Vell. (Meliaceae), two endangered native species of subtropical rainforests in Argentina. Both species produced desiccation-tolerant seeds, independently of collection year, seed traits, or climatic conditions. Depending on the species, seed traits and longevity varied across years and showed strong relationships with temperature and precipitation, particularly during seed development. *Cedrela balansae* seeds are medium-lived seeds and have high longevity under standard seed banking conditions, suggesting strong potential for long-term ex situ conservation. *Cedrela fissilis* seeds are short-lived seeds and have high sensitivity to the storage environment. Correlations among climatic variables and seed traits and longevity parameters suggest that future warming and drying environments may shorten the window for germination and seedling establishment, with species-specific responses depending on climatic conditions during seed development. These results highlight the importance of climate effects in determining seed traits and seed longevity and emphasize the role of seed banking as a critical conservation strategy under climate change.

## 1. Introduction

Global climate change is affecting plant regeneration from seeds, which may compromise population recruitment and, in the long term, threaten species persistence [1,2]. In Argentina, recent decades have exhibited significant trends in temperature extremes, more frequent heatwaves, increased precipitation, and severe droughts [3]. These climatic changes have, among other consequences, driven substantial variations in forest dynamics and reductions in mean annual net primary productivity [4]. In addition, nearly three million hectares of forest have been lost, positioning Argentina among the ten countries with the highest rates of recent forest loss [4]. This substantial reduction in forest area has been primarily driven by land use change, particularly the expansion of agriculture and grazing, and anthropogenic fires. As a result, the diversity of native forests has declined, and numerous species of high economic value have suffered genetic erosion, local extinction, or are considered endangered or threatened at regional and/or global scales [5,6]. These cumulative threats emphasize the importance of coordinated actions for storing seeds of forest species to guarantee their use in breeding, plant propagation, and ecological restoration of ecosystems [7].

Ex situ seed conservation in germplasm or seed banks is widely recognized as the most efficient and cost-effective strategy for long-term preservation of seeds of many plant species, since it safeguards genetically and geographically representative samples for future use [8,9]. To achieve effective conservation, it is essential to determine seed desiccation tolerance, storage behavior, and longevity [10]. Based on seed desiccation tolerance (DT) and storage behavior, seeds are classified into three categories: orthodox, where seeds can tolerate drying to a moisture content (MC) of approximately 3–7% and subzero temperature storage (−20 °C); recalcitrant, where seeds are sensitive to both desiccation and subzero temperatures; and intermediate, where seeds are sensitive to drying below 10% MC and/or to subzero temperatures [11]. DT has been associated with ecological, physiological, and morphological seed traits [12,13,14,15,16]. Tropical species are more likely to produce desiccation-sensitive seeds than temperate ones, and species with large seeds, thin coats, and high MC at dispersal time are more prone to desiccation than small seeds with thick coats and low MC. However, this classification is based on seed behavior at full maturity, before germination begins, and for many species, the transition from maturation to germination is not clearly defined, complicating seed classification [17]. In this sense, Walters [18] proposed viewing seed responses to desiccation as a continuum—from fully desiccation-tolerant to highly desiccation-sensitive—rather than as discrete categories, highlighting the need for quantitative evaluation of each seed lot.

Seed longevity is defined as the period during which seeds maintain viability at or above a target threshold under air-dry storage conditions [19]. In orthodox seeds, longevity increases as both temperature and seed MC decrease [20]. Consequently, long-term storage of orthodox seeds under low MC and subzero temperatures is the most widely used method for ex situ conservation of plant genetic resources. To estimate and to compare seed longevity in seed banks, accelerated (controlled) ageing tests—conducted at high MC and temperature—are commonly used [21,22,23,24]. Thus, loss of seed viability during accelerated aging or storage can be predicted by the seed viability equations [20]:

*v* = *K_i_* − (*p*/*σ*) (1)

where *v* is viability (in normal equivalent deviates, NED) of the seed lot after *p* days in storage, *K_i_* is the initial viability (NED) of the seed lots, *σ* is the time (d) for viability to decrease by 1 NED (i.e., the standard deviation of the normal distribution of seed deaths over time), and *σ*^−1^ is the slope of the transformed survival curve.

The effects of temperature (*t*) and MC (*m*) on seed longevity are species-specific, according to the following equation:

log_10_ *σ* = *K_E_* − *C_W_* log_10_ *m* − *C_H_ t* − *C_Q_ t*^2^ (2)

where *K_E_* is a species-specific constant for inherent seed longevity; *C_W_* is species-specific

MC quantifies the logarithmic relationship between seed longevity (*σ*) and seed MC (*m*); *C_H_* and *C_Q_* are species-specific temperature constants for the quadratic relationship between seed longevity and storage temperature, *t*, respectively.

Combining Equations (1) and (2) produces the full viability model, i.e., Equation (3):

*ν* = *K_i_* − *p*/10*^KE^*
^− *CW* log10 *m* − *CH t* − *CQ t*2^ (3)

Considering that the effects of temperature on longevity appear to be the same for all species, *C_H_* and *C_Q_*, take universal values of 0.0329 and 0.000478, respectively [25].

Seed longevity also has been linked to the climate of the area where seeds of the species were collected as well as to seed traits [9,24]. In general, seeds from species inhabiting warmer and drier regions exhibit greater longevity than those from cooler and wetter environments, and small-seeded and non-oil-rich species tend to show longer lifespan than large-seeded or oil-seeded species [15,16,24,26]. Nevertheless, correlations between seed traits and longevity are not consistent across all species [9,24]. In addition, intraspecific and interannual variation in seed longevity have been associated with local environmental conditions during seed development [27,28,29,30]. For example, White [30] showed that accessions collected from the same alpine population for several years differed in longevity, with seeds developed in wetter years being shorter-lived than those from drier years. However, few studies have investigated seed DT, storage behavior, and longevity in native tree species, probably due to their inherent longevity and responses to MC and temperature being less uniform than in crops [26].

The genus *Cedrela* (Meliaceae) is distributed from Mexico (24° N) to Argentina (28° S) and comprises tropical and subtropical species [31], being a protected genus [5]. In Argentina, four *Cedrela* species have been reported, all occurring in the northwestern and northeastern regions of the country. Thus, *C. angustifolia*, *C. balansae*, and *C. saltensis* are distributed at the different altitudinal levels of the Yungas Rainforest ecoregion in the northwest, while *C. fissilis* is present in the Paranaense Rainforest ecoregion in the northeast. *Cedrela balansae* and *C. fissilis* are considered important forest resources due to their fast development in forest enrichment systems and their quality timber, making them the most commercially valuable species in both local and international markets [32]. As a result, they have been subjected to intensive logging, which has led to reductions in genetic diversity and alterations in population structure [33,34,35]. At present, *C. balansae* is classified as endangered and *C. fissilis* as vulnerable species [5]. Mayrinck [6] also cited *C. fissilis* as an endangered species for Cerrado and Atlantic Forest Biome of Brazil. In addition, *C. fisilis* also is included in CITES (https://cites.org/eng/app/appendices.php; accessed on 30 December 2025).

*Cedrela balansae* C. DC. and *C. fissilis* Vell. are long-lived, deciduous canopy trees that can reach heights of up to 20 m. Flowering of *C. balansae* occurs between November and December, whereas that of *C. fissilis* is from September to December. Fruiting begins immediately after flowering, and capsules require approximately 6–8 months to reach maturity. Fruit development extends until August for *C. balansae* and July for *C. fissilis*, when the dehiscent capsules mature and release numerous winged seeds that are primarily dispersed by wind during the dry season [36,37].

At present, ex situ conservation of these species is primarily achieved through living collections established in clonal banks and clonal seed orchards [38]. However, the growing demand for seeds to support propagation, breeding, reforestation, and ecological restoration programs highlights the need for additional conservation strategies. While living collections can provide seeds in the short term, seed banks are essential as a complementary approach to safeguard genetic variability, particularly in the face of changing climatic conditions. Species of *Cedrela* are reported to have orthodox seeds [39,40], but Silva et al. [39] found that germination and vigor of *C. fissilis* seeds were greatly reduced during storage. Identifying seed desiccation tolerance, storage behavior, longevity, and temporal variation in these two *Cedrela* species is essential for prioritizing immediate post-harvest processing, selecting appropriate storage techniques, and planning viability monitoring and regeneration cycles.

Over the last two decades, collection sites (populations) of *C. balansae* and *C. fissilis* have been experiencing significant increases in temperatures and decreases in precipitation (Figure S1). Thus, we examined interannual variation in seed traits, desiccation tolerance, storage behavior, and longevity of these two species and their association between each other and with climatic conditions of seed provenance. Additionally, we predict longevity under different storage conditions of MC and temperature using the viability equation. For this, we used three accessions collected from the same population for each species between 2013 and 2016. Specifically, we hypothesized that interannual variation in seed traits and/or in climatic conditions affect desiccation tolerance, storage behavior, and/or seed longevity. We expected that seeds with lower seed mass, MC or lipid content, and/or produced in warmer and drier years would show higher tolerance to desiccation and longevity than seeds with higher mass, MC or lipid content, and/or produced in cooler and moist years.

## 2. Results

### 2.1. Seed Traits

Seed mass, lipid content, viability, MC, and/or germination percentage (GP) of fresh seeds varied across years, depending on the species (Table 1). In *C. balansae*, seeds collected in 2013 showed lower seed mass, viability, MC, and GP than those collected in the other years, while lipid content did not vary among years. Seeds of *C. fissilis* collected in 2013 showed lower seed mass and MC than in the other years, whereas lipid content in 2014 was higher than in the other years. Viability and GP did not vary over the years. For both species, the time to 50% germination of viable seeds (*t*_50_) did not vary among years, being on average 7.38 d for *C. balansae* and 7.78 d for *C. fissilis*.

### 2.2. Seed Desiccation Tolerance

For both species, MC, GP, and *t*_50_ varied significantly among study years and/or treatments (Table 1). For *C. balansae*, the highest MC values were for seeds collected in 2016, while GP was higher in 2015 and 2016 than in 2013. No significant differences were detected in *t*_50_ or VLI among years for any treatment. For all years of study, MC was significantly lower in the LiCl treatment than in the other treatments. In 2013, no significant differences in GP or *t*_50_ were registered among treatments, whereas in 2015 and 2016, GP was lower and *t*_50_ higher in seeds hydrated with KNO_3_ compared to other treatments. In 2013, VLI did not vary between treatments, whereas in 2015 and 2016 VLI values were significantly higher in KNO_3_ than in the other treatments.

For *C. fissilis*, MC was higher in 2014 in CaNO_3_ than in the other treatments. No significant differences in GP or VLI were registered among years for any treatment, whereas *t*_50_ was lower in 2014 than in the other years across all treatments. In all study years, MC was significantly lower in the LiCl treatment than in the other treatments. GP decreased significantly after drying with LiCl and after hydration with CaNO_3_, NaCl, and KNO_3_, with the lowest values registered in KNO_3_. The *t*_50_ values increased with increasing MC, reaching their highest values in KNO_3_ in 2014 and 2016 and in NaCl in 2016. In 2013, VLI did not vary between treatments, whereas VLI values were significantly higher in KNO_3_ in 2015 and 2016 and in NaCl in 2016.

### 2.3. Seed Storage Behavior

**Figure 1:**
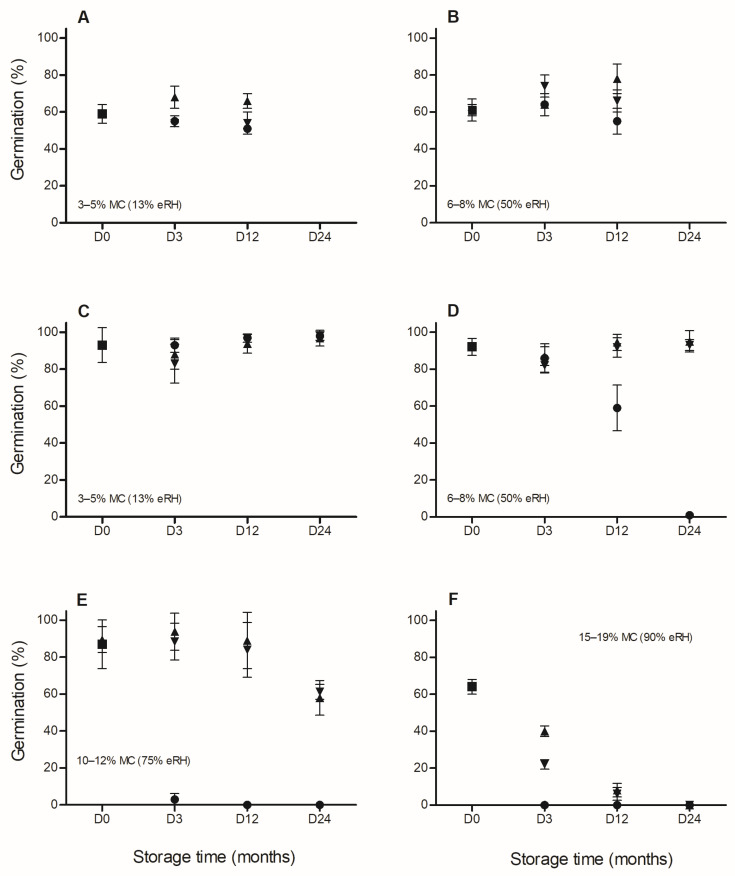
Germination (%) after desiccation with saturated salt solutions of LiCl (**A**,**C**) and hydration with CaNO_3_ (**B**,**D**), NaCl (**E**), and KNO_3_ (**F**) to moisture content (MC) shown at each graph (D0: ■) and after subsequent hermetic storage at −20 °C (▲), 5 °C (▼), and 25 °C (●) for 3, 12, and 24 months (D3, D12, and D24, respectively) in 2013 (**A**,**B**) and 2015 (**C**–**F**) for *Cedrela balansae*. Each value of germination is the mean (±SE).

**Figure 2:**
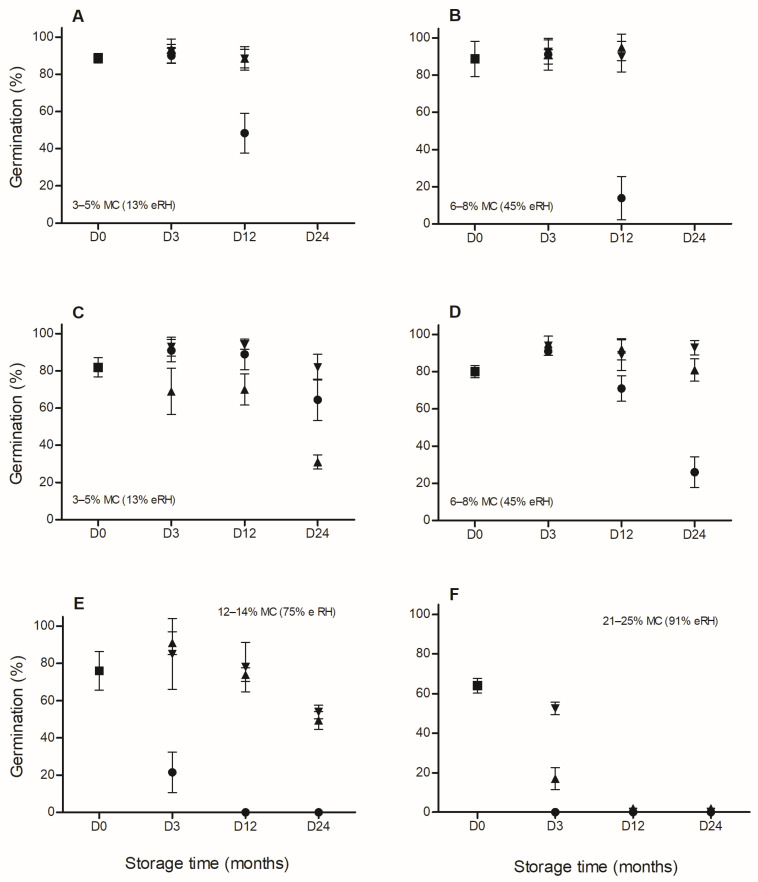
Germination (%) after desiccation with saturated salt solutions of LiCl (**A**,**C**) and hydration with CaNO_3_ (**B**,**D**), NaCl (**E**), and KNO_3_ (**F**) to moisture content (MC) shown at each graph (D0: ■) and after subsequent hermetic storage at −20 °C (▲), 5 °C (▼), and 25 °C (●) for 3, 12, and 24 months (D3, D12, and D24, respectively) in 2013 (**A**,**B**) and 2014 (**C**–**F**) for *Cedrela fissilis*. Each value of germination is the mean (±SE).

For both species, seed storage behavior under different conditions of MC and temperature varied between years (Figure 1 and Figure 2). Thus, for *C. balansae*, in 2013, GP and *t*_50_ were not significantly affected by MC, temperature, storage period, or their interactions (*p* > 0.05; Figure 1A,B). In contrast, GP in 2015 was significantly affected by all these factors and their interactions (*p* < 0.05; Figure 1C–F). At 25 °C, GP was significantly reduced after 12 months in seeds stored with 6–8% MC and after 3 months in seeds stored with 10–12% MC. At 5 °C and −20 °C, significant decreases in GP were detected after 24 months in seeds with 10–12% MC and after 3 months in seeds with 15–19% MC, respectively. No germination (dead seeds) was recorded at 25 °C after 24, 12, and 3 months in seeds stored with 6–8%, 10–12%, and 15–19% MC, respectively, or at 5 °C and −20 °C after 24 months in seeds with 15–19% MC. The *t*_50_ values were significantly higher in seeds stored at 15–19% MC across all storage temperatures than in the other treatments (on average 11 vs. 6–8 days, respectively).

For *C. fissilis*, GP and *t*_50_ in 2013 were significantly affected by temperature, storage period, and temperature × storage period and MC × temperature × storage period interactions (*p* < 0.05; Figure 2A,B). Thus, GP at 25 °C was significantly lower after 12 months in seeds stored with 3–5% and 6–8% MC, and *t*_50_ was higher (8 vs. 6 days) than in the other treatments. In 2014, GP and *t*_50_ were significantly affected by all these factors and their interactions (*p* < 0.05; Figure 2C–F). At −20 °C, GP decreased significantly after 24 months in seeds stored with 3–5% and after 3 months in seeds with 21–25% MC. At 25 °C, GP was significantly reduced after 3 and 24 months in seeds stored with 12–14% and 6–8% MC, respectively, and no germination (dead seeds) was recorded after 3 and 12 months in seeds stored with 21–25% and 12–14% MC, respectively. At 5 °C and −20 °C, significant decreases in GP were detected after 24 months in seeds with 12–14% MC, and no GP was registered after 12 months in seeds with 21–25% MC. The *t*_50_ values were significantly (*p* < 0.05) higher in seeds stored at 21–25% MC across all storage temperatures than in the other treatments (on average 10 vs. 6–7 days, respectively).

### 2.4. Seed Longevity

#### 2.4.1. Comparative Longevity

For both species, seed longevity parameters significantly varied between study years (*p* < 0.05; Table 2). For *C. balansae*, *K_i_*, *σ*^−1^ and *p*_50_ were higher in 2015 than in 2013, whereas *σ* was higher in 2013 than in 2015. For *C. fissilis*, *K_i_* did not differ significantly between years, but *σ* and *p*_50_ were higher and *σ*^−1^ lower in 2014 than in 2013.

#### 2.4.2. Estimation and Validation of C_W_ and K_E_ Constants

**Figure 3:**
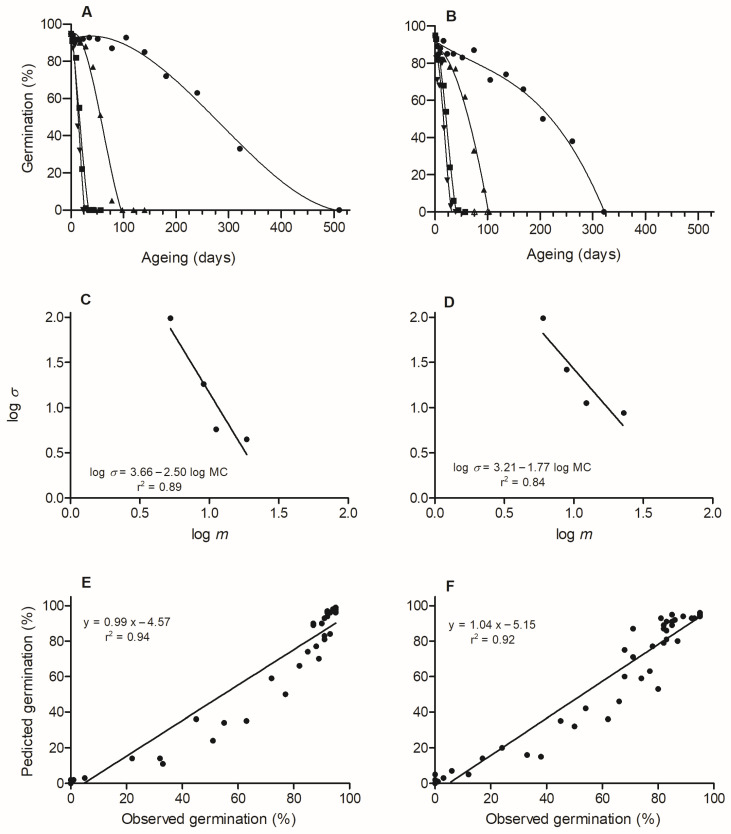
Survival curves (% germination versus ageing period) fitted by probit analysis for *Cedrela balansae* (**A**) and *C. fissilis* (**B**) seeds stored at relative humidities of 45% (●), 60% (▲), 75% (■), and 88% (▼) at 35 °C. Regression analyses showing the relationship between log_10_ (*σ*) and log_10_ (moisture content, *m*), and between observed and predicted germination (%) for *Cedrela balansae* (**C**,**E**) and *C. fissilis* (**D**,**F**).

For both species, seed survival curves exhibited a typical sigmoid pattern on the percentage scale, with seeds at 45% eRH (5.2–6% MC) showing a longer lag period, followed by seeds at 60% eRH (8.9–9% MC) and finally by those at ≥75% eRH (≥11.2% MC; Figure 3A,B). For both species, there was no significant difference in *K_i_* among MC, being on average 2.22 for *C. balansae* and 1.66 for *C. fissilis* (Table 2). However, mean values of *σ*, *p*_50_, and *σ*^−1^ differed significantly between MC (*p* < 0.05), with both *σ* and *p*_50_ decreasing and *σ*^−1^ increasing as MC increased. A negative relationship was observed between seed longevity (log *σ*) and MC (log *m*; Figure 3C,D).

The estimated *K* and *C_W_* values were 3.6618 (s.e. = 0.0409) and 2.4983 (s.e. = 0.0395), respectively, for *C. balansae* and 3.2039 (s.e. = 0.0419) and 1.7706 (s.e. = 0.0489), respectively, for *C. fissilis*. Using these *K* values and the universal constants *C_H_* (0.0329) and *C_Q_* (0.000478), the derived *K_E_* values were 5.3988 ± 0.0409 for *C. balansae* and 3.8677 ± 0.3680 for *C. fissilis*. A positive relationship was observed between predicted GP by the viability equation and observed GP of storage behavior tests (Figure 3E,F), which validated the estimated constants.

#### 2.4.3. Prediction of Seed Longevity Under Different Storage Conditions

Predictions of seed longevity (*p*_50_) indicated that seeds stored with MC > 6.8% at −20 °C ranged between 16 and 27 years for *C. balansae* and between 2 and 3 years for *C. fissilis* (Table 3). In contrast, when stored at ambient room temperature (22 °C), *p*_50_ values were ≤1 year for *C. balansae* and <2 months for *C. fissilis*. However, under standard seed bank conditions (3–5% MC at −20 °C), *C. balansae* seeds showed a predicted *p*_50_ of 115 years, with a *σ* of 67 years. For *C. fissilis*, predicted *p*_50_ under the same conditions was only 7 years, with a *σ* of 4 years.

#### 2.4.4. Associations Between Seed Traits, Desiccation Tolerance and Longevity Parameters, and Climatic Variables of Seed Provenance

For both species, Spearman’s correlation analysis revealed significant associations among seed traits, longevity parameters, and/or climatic variables, whereas lipid content and VLI were not correlated with any of the variables considered (*p* < 0.05; Table 4). In *C. balansae*, seed mass, MC, *K_i_*, and *p*_50_ were positively intercorrelated, while *σ* was negatively correlated with these variables. These traits also were positively associated with both precipitation variables (APP and PP_J-J_) and negatively associated with temperature variables, including annual temperatures (MaAT and MeAT) and temperatures during seed development (MaT_J-J_, MeT_J-J_, and MiT_J-J_). Conversely, *σ* showed negative correlations with both precipitations and positive correlations with all these temperature variables. In *C. fissilis*, only seed mass was positively correlated with MC. Seed mass, *K_i_*, and *p*_50_ were positively correlated with all annual temperature variables. *K_i_* and *p*_50_ also were positively correlated with both precipitations and negatively with temperatures during the seed development period.

## 3. Discussion

Temporal variation in seed traits and functional responses has received comparatively less attention than inter- and intraspecific variability [27]. However, understanding how seed mass, desiccation tolerance, storage behavior, and longevity vary among years is essential for effective seed bank management [42] and for predicting natural regeneration of species in environments increasingly affected by climate change [26,30,43]. In this study, our hypothesis was partially supported, i.e., interannual variation in seed traits and in the climatic conditions of seed provenance did not affect seed desiccation tolerance in *C. balansae* and *C. fissilis*, but it did influence seed storage behavior and longevity.

### 3.1. Seed Traits

Environmental conditions experienced by mother plants strongly influence seed development and, consequently, seed traits [44,45]. High temperatures and/or reduced water availability during seed development frequently affect seed mass, MC, dormancy, and germination, although responses are not always consistent across species [12,46]. In both *Cedrela* species, the lowest seed mass and MC were recorded in 2013, coinciding with lower accumulated precipitation during seed development compared with other years. Similar patterns have been reported in alpine species, where seed mass is more closely linked to soil water availability than to temperature [47]. In *C. balansae*, seed viability and germination also were reduced in 2013, whereas in *C. fissilis*, no interannual differences in these traits were found. This apparently neutral effect of warmer and drier maternal environments on seed viability and germination is consistent with previous reports for threatened *Acacia* species in Western Australia [27] and alpine Australian species [48].

Our findings have important implications for the resilience of both species under changing climatic conditions. In *C. balansae*, smaller-winged seeds may favor dispersal to new habitats, but their reduced viability and germination could limit seedling recruitment. In contrast, stability of seed viability and germination in *C. fissilis* may confer an advantage under changing environmental conditions and during colonization of new habitats.

### 3.2. Seed Desiccation Tolerance

Seed mass and MC at shedding are commonly used predictors of desiccation tolerance [11,15,49], with desiccation-tolerant seeds typically having a thousand-seed weight (TSW) < 2500 g and MC ≤ 23% [11]. In addition, in environments with dry seasons, such as in the Yuto and El Naranjo localities, most species had low seed MC (<20%), since they usually tolerate dry periods better than those with high MC [14,50,51]. In this case, although seed mass and MC varied among years, seeds of both species consistently showed low TSW (20–27 g) and low MC (5.5–7.5%) and were dispersed in the dry season (in winter months, July and August), strongly indicating desiccation tolerance.

In *C. balansae*, no differences in germination between fresh and dried seeds (3–5% MC) were detected, and viability loss index (VLI) values were ≤0.05, confirming desiccation tolerance. In *C. fissilis*, drying reduced germination by 9–14%, with VLI values between 0.09 and 0.15, classifying the seeds as potentially desiccation-tolerant [49]. Similar partial reductions in germination after drying have been reported for other tree species [52,53], supporting the idea of a continuum in desiccation tolerance rather than discrete categories, in which some species and/or populations are more susceptible to desiccation than others [18]. On the other hand, seed hydration above 18% MC reduced germination and increased *t*_50_, probably due to enhanced metabolic damage and fungal proliferation [42]. Nevertheless, even under these conditions, VLI values remained within the range of potential desiccation tolerance, reinforcing the overall orthodox behavior of both species.

### 3.3. Storage Behavior

Standard seed banking protocols involve drying seeds to 3–7% MC and storing them at −20 ± 2 °C (base collection) or 5 ± 2 °C (active collection) [10]. While effective for most crops, wild tree species often show heterogeneous responses to storage conditions [26]. Moreover, storage behavior is frequently inferred from single-seed lots, limiting broader inference. In our study, germination and *t*_50_ were influenced by seed MC, storage temperature, storage duration, and collection year, with species-specific responses. In *C. balansae*, seeds dried to 3–5% MC consistently maintained viability across all storage temperatures, whereas seeds with 6–8% MC showed year-dependent responses, with seeds collected in 2015 remaining viable only when stored at 5 and −20 °C. In *C. fissilis*, seeds with both 3–5% and 6–8% MC maintained viability at 5 and −20 °C in both years; however, seeds collected in 2014 with 3–5% MC lost viability at −20 °C after 24 months of storage. This loss of viability was probably associated with the higher lipid content of these seeds, consistent with reports of lipid crystallization damage during freezing in oily seeds [54].

On the other hand, seeds stored at MC > 10–12% retained viability for up to 1 year at low temperatures but deteriorated thereafter, while seeds with MC > 15–21% lost viability rapidly, probably due to physical damage caused by lethal ice formation, reinforcing the advice against storing seeds with high MC [55]. At 25 °C, viability loss at MC ≥ 6% was attributable to physiological ageing processes [56], including lipid peroxidation, as previously described for *C. fissilis* [39,57].

Overall, both species can be classified as orthodox, in agreement with previous reports [6,40,41,58]. However, for *C. fissilis*, lipid content should be carefully considered prior to long-term storage at −20 °C, since viability may decrease if seeds are stored at −20 °C. Hamilton et al. [16] suggested that for oily seeds with variable responses and storage limitations, cryopreservation is the most reliable long-term conservation strategy. For this species a preliminary study showed that cryopreservation of seeds tolerated freezing in LN without any loss in viability when MC was in the range of 3–5%, indicating that this technique would be a reliable strategy. Further studies are needed to define the optimal hydration window for cryogenic storage, which can be very wide for orthodox seeds [59].

### 3.4. Seed Longevity

Seed longevity is central to seed bank management for planning viability monitoring intervals and regeneration cycles, and it is also key to understanding soil seed persistence [60]. In our study, accelerated ageing tests revealed interannual variation in longevity, with both species showing the lowest *p*_50_ values in seeds collected in 2013. White et al. [30] demonstrated that such interannual variation in seed longevity may arise from differences in *K_i_*, in the time required for viability to fall one NED (*σ*), or from variation in both parameters. Consistent with this, the reduced *p*_50_ observed in *C. balansae* was primarily associated with lower initial viability (*K_i_*), whereas in *C. fissilis* it was mainly linked to lower *σ* values. Because *p*_50_ is inherently dependent on *K_i_*, *σ* has been proposed as a more appropriate measure of intrinsic seed longevity, since it is independent of initial seed quality [61]. Based on *p*_50_ values ranging from 28 to 47 days, seeds of both *Cedrela* species would be classified as medium-lived (*p*_50_ > 10 to ≤100 d) [22], similar to what has been reported for other tree species [21,53]. However, relative longevity classifications are valid only under the specific ageing conditions under which they are derived [54]. For example, these authors reported *σ* values of 30.21 days for *Xanthorrhoea preissii* seeds aged at 35 °C with 10% MC but only 6.63 days when seeds were aged at 45 °C with the same MC. Therefore, additional ageing tests conducted under standardized conditions (45 °C and 60% RH) would be required to determine more robustly whether the *Cedrela* species should be classified as medium- or short-lived.

The seed viability equation can provide accurate predictions of seed longevity when applied within a valid range of conditions, and it is widely used to describe how orthodox seeds lose viability during storage under different combinations of seed MC and temperature [20,23,54,62]. However, reliable application of this model requires an estimation of species-specific constants [20]. In this study, we used four MC levels at 35 °C to estimate these constants and associated longevity parameters. For both species, the constant *K_i_* did not differ significantly among storage environments, reflecting the use of a single-seed lot and satisfying a key assumption of the seed viability equation [20]. Survival curves differed among MC treatments, with seeds stored at lower MC exhibiting greater longevity (higher *p*_50_) than those stored at higher MC. Consistent with this pattern, a negative relationship was observed between seed longevity (log *σ*) and seed MC (log *m*), highlighting the importance of seed drying for prolonging longevity. The estimated *C_W_* constant, which describes the relative effect of MC on longevity, was 2.4983 for *C. balansae* and 1.7706 for *C. fissilis*. These values are lower than those reported for *C. odorata* (*C_W_* = 3.80) [62], possibly reflecting its higher seed oil content (21%) compared with our *Cedrela* species (13–19%), although relationships between oil content and *C_W_* are not consistent across studies [23]. The *K_E_* constant, which represents inherent seed longevity [20], was estimated at 5.3988 for *C. balansae* and 3.9729 for *C. fissilis*. These values also are lower than those reported for *C. odorata* (*K_E_* = 6.90) [62], indicating higher inherent longevity in *C. odorata*, followed by *C. balansae* and then *C. fissilis*. Moreover, the *C_W_* and *K_E_* constants were validated using independent data from the storage behavior experiments, confirming their suitability for predicting longevity in other seed lots of each species.

Identifying the period during which seed viability can be maintained under different storage conditions is particularly relevant for forest researchers working with species that have abundant seed production in some years and scarcity in others, as is the case for *C. balansae*. Using the viability tools of the Seed Information Database [41] together with our estimated constants, we assessed the potential longevity of seeds stored under different combinations of MC and temperature. *C. balansae* exhibited high longevity, with a *p*_50_ on the order of hundreds of years and a loss of one NED of viability occurring after 67 years, indicating strong potential for long-term ex situ conservation. In contrast, *C. fissilis* showed much lower longevity, with a *p*_50_ of around 7 years and a comparable decline in viability occurring after approximately 4 years, highlighting its greater sensitivity to temperature and MC during storage despite being desiccation-tolerant. Moreover, under uncontrolled ambient conditions (≈22 °C and MC > 6%), both *p*_50_ and *σ* declined sharply, reaching 1 year or less for *C. balansae* and less than 2 months for *C. fissilis*. According to these estimations, and consistent with results from comparative longevity tests, *C. balansae* can be classified as having medium-lived seeds, whereas *C. fissilis* appears to have short-lived seeds, contrary to comparative longevity test classification but in agreement with reports for this species by Carvalho [63]. Furthermore, our results for *C. fissilis* support the hypothesis proposed by Long et al. [43] that seed lifespan estimated under laboratory conditions reflects persistence under natural conditions, since this species also has been reported to form a transitory soil seed bank [64].

### 3.5. Associations Between Seed Traits, Desiccation Tolerance, and Longevity Parameters and Climatic Variables of Seed Provenance

Climate change is increasingly influencing tree reproductive processes by altering phenology, seed production, seed functional traits, and seedling establishment, with potential long-term consequences for species dispersal, persistence, and conservation [1,2]. Our correlation analyses showed that lipid content for both species was not associated with other seed traits, including desiccation tolerance and seed longevity, or with climatic variables, in agreement with previous findings across a wide range of species [9,24]. Furthermore, seed desiccation tolerance, estimated using the viability loss index (VLI), was not related to seed traits, seed longevity, or climatic variables, suggesting that desiccation tolerance may be a heritable trait and therefore species-specific [13].

Conversely, for *C. balansae*, seed mass and MC were positively associated with each other and with precipitation and negatively associated with temperature, both at the annual scale and during seed development. These results are consistent with those of Cochrane [27] and Vázquez-Ramírez and Venn [48], who reported that seed mass tends to decrease under warmer and drier environmental conditions. Regarding seed longevity, both *K_i_* and *p*_50_ were positively associated with seed mass, MC, and precipitation and negatively associated with temperature (both at the annual scale and during seed development). These trends contrast with findings from other studies reporting that larger seeds and/or seeds produced in warmer and drier years exhibit lower *p*_50_ values than smaller seeds and/or seeds produced in cooler and wetter years [9,24,30]. In contrast, *σ* showed an inverse pattern, increasing with temperature and decreasing with precipitation, suggesting that seeds produced under warmer and drier conditions may exhibit greater resistance to aging than those produced in colder and wetter years. This pattern is consistent with Mondoni et al. [29], who demonstrated that warmer conditions during seed development enhanced resistance to aging in alpine species, supporting the idea that seed longevity may exhibit adaptive responses under future warming scenarios. As proposed by these authors, adaptive adjustments in seed longevity driven by transgenerational plasticity may therefore play a fundamental role in species survival and persistence under future environmental challenges, such as climate warming.

In contrast, for *C. fissilis*, seed mass was the only trait positively correlated with MC and with annual temperature, a pattern similar to that reported for some alpine species [65]. In addition, *K_i_* and *p*_50_ were positively correlated with annual temperature and precipitation (both at annual scale and during seed development) but negatively correlated with temperatures during seed development period. These results indicate that the thermal conditions experienced by maternal plants during the reproductive phase are particularly critical for determining seed longevity. In this context, several studies have emphasized that environmental conditions during seed development are decisive in the determination of seed longevity [2,24,30]. Similarly, the positive relationship between seed longevity and precipitation is consistent with findings by Merritt et al. [66] for Australian species; however, the mechanisms linking precipitation to seed longevity remain poorly understood and require further investigation [30]. However, if we consider that *C. fissilis* is naturally distributed in the subtropical Paranaense Rainforest (Atlantic Forest) of northeastern Argentina, which is characterized by a humid subtropical climate without a dry season, an average annual rainfall of approximately 2.000 mm, and a mean annual temperature of 20.2 °C, it is therefore not surprising that seed traits and longevity increased with temperature and precipitation and that temperature during seed development can be a critical factor influencing initial seed quality and longevity. Further research, including seed collections from populations across the species’ natural distribution, is required to establish the relative contributions of genetic and environmental factors in determining seed traits and longevity.

Therefore, as climate change progresses with increasing temperatures and decreasing precipitation, the window of opportunity for successful germination and seedling establishment is likely to become shorter for both species, as has been reported for other tree species [67]. For *C. balansae*, reductions in seed mass, MC, initial seed quality, and longevity are likely to negatively affect seed germination and seedling establishment; however, an increase in seed deterioration (*σ*) may enhance seed persistence in the soil, thereby increasing the likelihood of germination when environmental conditions become favorable. In contrast, *C. fissilis* may remain unaffected or may even exhibit an increase in seed mass, initial seed quality, and longevity under higher annual temperatures; however, if temperatures increase and precipitation decreases during seed development, both initial seed quality and longevity are expected to decline, reducing seed persistence in the soil and limiting germination and seedling establishment. In this context, these findings highlight the importance of ex situ conservation through seed banking for both species.

## 4. Materials and Methods

### 4.1. Seed Collection

**Figure 4:**
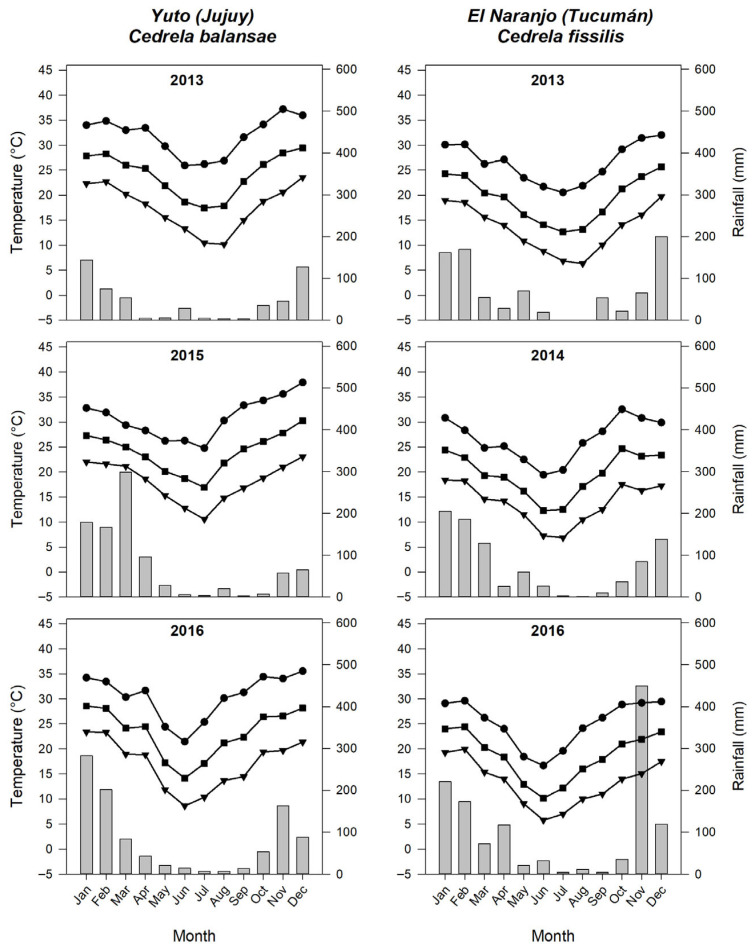
Monthly mean (■), maximum (●), and minimum (▼) temperatures and accumulated rainfall (grey bars) of Yuto locality, Jujuy province (*Cedrela balansae*), and of El Naranjo locality, Tucumán province (*Cedrela fissilis*).

Mature seeds were collected at the end of July for *C. balansae* and June for *C. fissilis* from 20 individuals per species in two seed production stands located in the northwestern provinces of Argentina (Jujuy and Tucumán). Seeds of *C. balansae* were obtained in 2013, 2015, and 2016 from Yuto locality in the Jujuy province (23°38′ S, 64°28′ W; 349 m a.s.l.). Seeds of *C. fissilis* were collected in 2013, 2014, and 2016 from El Naranjo locality in the Tucumán province (26°39′ S, 65°02′ W; 665 m a.s.l.) as a seed-source provenance introduced from Guaraní reserve (26°55′ S, 54°13′ W; 482 m a.s.l.) in the Misiones province of Argentina. The climate in both collection sites is characterized by two well-defined seasons: a dry season from late April to September and a wet season during the remainder of the year, with more than 80% of annual precipitation occurring between October and March (Figure 4).

### 4.2. Seed Traits

For each species and year of collection, immediately after seed collection, seed mass, lipid and moisture content (%), viability and germination (%), and the time to 50% germination were determined in fresh seeds. The mass of 1000 seeds (TSW) was estimated by weighing five replicates of 100 seeds each with a precision balance to 0.0001 mg accuracy (Denver Instrument APX-200, Denver Instrument Company, Denver, CO, USA). Total lipid content was determined using a modified protocol [68]. Three pre-weighed samples of ground seeds (2.0 g) were extracted overnight with petroleum ether. Then, the solvent was removed via evaporation, and the extract was further purified by dissolving it in chloroform and filtering through Whatman No. 1 paper. The combined filtrate and washings then were dried and weight. The amount of lipids per gram of seed dry weight was calculated (expressed in % dry weight basis). Seed moisture content (MC) was determined gravimetrically by drying three replicates of 10 seeds each at 103 °C for 17 h (expressed in % fresh weight basis). Seed viability (%) was determined in 100 seeds using the tetrazolium chloride staining technique [69].

Seed germination was determined in four replicates of 25 seeds each for each year. Seeds were sown in Petri dishes on two sheets of filter paper moistened with distilled water and incubated in a chamber at 25 °C with a photoperiod of 12 h light/12 h darkness (optimal conditions). The number of germinated seeds (i.e., with radicle emergence) was recorded daily for 30 days. At the end of all germination tests, when no additional germination had occurred for 2 weeks, a cut test was conducted to determine the viability of the non-germinated seeds (soft or firm, i.e., dead or viable, respectively). The number of viable seeds per replicate was the number of firm non-germinated seeds + the germinated seeds. The final germination percentage (GP) was calculated based on the total number of viable seeds. The accumulated germination data were adjusted using the sigmoidal dose–response equation, and time to 50% germination of viable seeds (*t*_50_) for each treatment was estimated by linear interpolation between points on the germination curve adjacent to 50% germination.

### 4.3. Seed Desiccation Tolerance

In 2013, for both species, two sub-lots of 130 seeds each were placed in tulle bags and stored inside a sealed glass container (desiccator) at ambient temperature (22 °C), and MC was adjusted by drying over saturated solutions of LiCl (13% equilibrium relative humidity, eRH) to 3–5% MC and CaNO_3_ (50% eRH) to 6–9% MC [51]. The eRH inside each desiccator was monitored weekly using dataloggers (HOBO Temp/RH logger UX100-011, USA). After approximately 4 weeks, when the eRH of each desiccator was stabilized, GP and MC were determined. GP of fresh (untreated) seeds was used as a control. In 2014 for *C. fissilis* and in 2015 for *C. balansae*, two additional salt solutions were included: NaCl (75% eRH; 10–14% MC) and KNO_3_ (91% eRH; 15–25% MC), following the procedure described for LiCl and CaNO_3_.

Seed desiccation tolerance was estimated using the viability loss index (VLI) [49]:

VLI = (Fresh germination% − Dry (with LiCl) germination%)/Fresh germination%

VLI values theoretically vary from 0 (desiccation-tolerant seeds) to 1 (desiccation-sensitive seeds). Thus, seeds with VLI > 0.95 were considered as desiccation-sensitive, with 0.95 > VLI > 0.5 as potentially desiccation-sensitive, with VLI < 0.05 as desiccation-tolerant, and with 0.05 < VLI < 0.5 as potentially desiccation-tolerant. In addition, we also estimated VLI for seeds submitted to CaNO_3_, NaCl, and KNO_3_ saturated salt solution to evaluate tolerance.

### 4.4. Seed Storage Behavior

In 2013, a factorial design experiment combining two MC levels (3–5 and 6–8%), three temperatures (−18, 5, and 25 °C), and two storage periods (3 and 12 months) was conducted for each species. Two sub-lots of 1700 seeds each were equilibrated in desiccators containing LiCl and CaNO_3_ and kept at 22 °C until the eRHs were reached. After approximately 4 weeks, MC and GP were determined for each seed lot. Then, 12 samples of 130 seeds each were sealed in aluminum-foil bags and stored at the specified temperatures [51]. In 2014 or 2015, depending on the species, two additional MC ranges were included (NaCl: 11–14% MC and KNO_3_: 15–25%MC). Accordingly, a factorial design was implemented combining four MC levels (3–5%, 6–8%, 10–14%, and 15–25%), three storage temperatures (−20°, 5°, and 25 °C), and three storage periods (3, 12, and 24 months). For each species, 5000 seeds were equilibrated in desiccators with LiCl, CaNO_3_, NaCl, and KNO_3_ and kept at 22 °C until eRHs were reached. After 4 weeks, MC and GP were determined, and 36 samples of 130 seeds each were sealed in aluminum-foil bags and stored at the specified temperatures. Each treatment (MC × temperature × storage period) was represented by one sample per bag. In all study years, MC and GP were evaluated for each treatment. 

### 4.5. Seed Longevity

#### 4.5.1. Comparative Seed Longevity 

In 2013, 2014, and 2015 (depending on the species), 11 samples of 100 seeds each were stored in tulle bags and placed into a desiccator with a saturated salt solution of NaNO_2_ (60% eRH) and kept in a chamber at 35 °C. One sample for each species was removed after each of 2, 5, 9, 20, 33, 54, 75, 100, 125, 140, and 152 days for germination testing. Germination data were plotted as a seed survival curve, where seed viability (GP) was plotted against ageing period. Time for viability to decline to 50% (*p*_50_) was estimated using the following equation:

*p*_50_ = *K_i_* × *σ* (4)

Species were ranked by *p*_50_ and assigned to the categories of short-lived (*p*_50_ > 1 and ≤10 days), medium-lived (*p*_50_ > 10 and ≤100 days), and long-lived (*p*_50_ > 100 and ≤1000 days), based on [22].

#### 4.5.2. Estimation and Validation of *C_W_* and *K_E_* Constants

In 2014 or 2015 (depending on the species), 14 samples of 100 seeds each were stored in tulle bags and placed into desiccators containing saturated salt solutions of CaNO_3_ (45% eRH), NaNO_2_ (60% eRH), NaCl (75% eRH), and KNO_3_ (88% eRH) at 35 °C. One sample per treatment was removed at scheduled intervals (10–14 dates) depending on how quickly seeds were losing viability and tested for germination. Loss of viability ranged between 42 days in KNO_3_ and 510 days in CaNO_3_ for *C. balansae* and between 39 days in KNO_3_ and 321 days in CaNO_3_ for *C. fissilis*.

The GP data from serial germination tests at the respective seed MC were fitted by probit analysis according to the viability equation (Equation (1)) [20]. This generated the fitted seed survival curves from which *K_i_*, *σ*, *σ*^−1^, and *p*_50_ were determined. To estimate *K_E_* and *C_W_* constants, the relationship between storage seed MC and seed longevity was quantified using linear regression, where log *σ* was plotted against the log of seed MC (*m*) to obtain the value of *C_W_* by fitting the following equation:

log *σ* = *K* − *C_W_* log *m* (5)

The value of *K_E_* was estimated by using the universal values of *C_H_* (0.0329) and *C_Q_* (0.000478) as well as *K* (Equation (3)) at ageing temperature (*t*) equal to 35 °C, as presented in the following equation:

*K_E_* = *K* + *C_H_ t* + *C_Q_ t*^2^ (6)

For validating the newly derived viability constants (*C_W_* and *K_E_*), we used the GP from the seed storage behavior experiments (see Section 2). Thus, we estimated initial viability (*K_i_*) of stored seeds under each combination of MC and temperature, with *C_W_* and *K_E_*, and with the viability equation. We predicted the GP for each combination and storage interval. The observed GP values were regressed against predicted GP values using linear regression.

#### 4.5.3. Prediction of Seed Longevity Under Different Storage Conditions

For both species, we used the estimated parameters of the seed viability equation to predict *σ* and *p*_50_ for a collection with an initial viability of 95% (1.64 NED) under a range of MCs and storage conditions. The MCs evaluated were the (1) MC of seeds at time of dispersal (e.g., 6.8% for both species), (2) MC of seeds equilibrated at standard seed bank drying conditions of 15% RH and 15 °C (*C. balansae*: 3.8% and *C. fissilis*: 4.3%), and (3) estimated MC of seeds equilibrated at ambient conditions with 50% RH and 22 °C (*C. balansae*: 8.3% and *C. fissilis*: 8.9%). Storage temperatures considered were −20 °C and 5 °C, standard seed bank storage conditions [10], and 22 °C (ambient room temperature of storage at clonal seed orchard facilities). Predictions were obtained using the viability tools available in the Seed Information Database [41].

#### 4.5.4. Climate Data

Climate information for the collection sites was sourced from NASA POWER. Maximum (Ma), mean (Me), and minimum (Mi) daily temperatures, as well as total precipitation (PP), were downloaded for the period 2000–2024. To assess long-term trends, daily data was aggregated to an annual level (MaAT, MeAT, MiAT, and APP; Figure S1). For correlation analyses, for each year of the study, MaAT, MeAT, MiAT, and APP and maximum, mean, and minimum temperatures and total precipitation (MaT_J-J_, MeT_J-J_, and MiT_J-J_, PP_J-J_, respectively) during seed development (January–July for *C. balansae* and January-June for *C. fissilis*) were also considered (Figure 4).

### 4.6. Statistical Analysis

General linear mixed models were used to analyze seed mass, lipid content, MC, and *t*_50_, *K_i_*, *σ*, *σ*^−1^, and *p*_50_ and to compare among years and/or treatments for each species. Variance heterogeneity was considered, and the AIC and BIC information criteria were used to select the best-fitted model. General linear mixed models for binomial distribution and logit link functions were used to explain variations in number of germinated seeds. As the number of viable seeds was not the same for all experimental units, this number was entered as a covariable. For all analysis, year, desiccation tolerance, and storage behavior treatment factors were considered as fixed effects. In all cases, all factor interactions were included in the models, and same information criteria were used to select the most parsimonious model, and the residual deviance/degrees of freedom ratio was calculated to assess if the goodness-of-fit of the model was reasonable and if there was over-dispersion. Treatments with no variance (none or all of the seeds germinated in each replicate) were excluded from the analyses. The post hoc DGC test of multiple comparisons of means was used to locate differences among factor levels or their combinations of means when the effect of the factor or their interaction was significant (*p* < 0.05). We explored the associations of seed traits (seed mass, lipid content, and MC), desiccation tolerance (VLI), and seed longevity parameters (*K_i_*, *σ*, and *p*_50_) between each other and with climatic variables (MaAT, MeAT, MiAT, AP, MaT_J-J_, MeT_J-J_, MiT_J-J_, and PP_J-J_), evaluating the significance of the Spearman correlation coefficient. All statistical analyses were conducted using the InfoStat software package (version 2017) [70].

## 5. Conclusions

This study demonstrates that interannual climatic variability during seed development plays a central role in shaping seed traits, storage behavior, and longevity in *C. balansae* and *C. fissilis*, with important implications under ongoing climate change. While both species produce desiccation-tolerant seeds, desiccation tolerance itself was largely independent of seed traits and climatic conditions, supporting the idea that it is primarily a species-specific characteristic. In contrast, seed mass, MC, initial seed quality, and longevity were strongly influenced by temperature and precipitation, particularly during the reproductive phase, highlighting the importance of maternal environmental effects on seed performance. Marked interspecific differences were observed in seed longevity and storage potential. *C. balansae* exhibited medium-lived seeds and comparatively high longevity under optimal storage conditions, indicating strong potential for long-term ex situ conservation, whereas *C. fissilis* showed short-lived seeds, much shorter longevity and greater sensitivity to storage environment, consistent with its transitory soil seed bank behavior. Correlations between climatic variables and longevity parameters suggest that future increases in temperature and reductions in precipitation may shorten the window for germination and seedling establishment in both species, although species-specific responses are expected depending on the timing of climatic stress. Overall, our findings highlight that seed longevity is a dynamic trait influenced by both environmental conditions and transgenerational plasticity, which may mediate species responses to future climate scenarios. Given the predicted shifts in temperature and precipitation regimes and the contrasting longevity observed between species and year of collection, ex situ conservation through seed banking represents a key strategy to safeguard genetic diversity and support the long-term persistence of these endangered forest species. Additionally, cryopreservation should be considered as a complementary approach for their long-term conservation. To our knowledge, this is one of the first studies to integrate interannual climatic variability, seed trait expression, and longevity parameters in subtropical tree species.

## Figures and Tables

**Table 1:** Seed mass (thousand-seed weight, TSW, g), lipid content (%), viability (%), moisture content (% MC), germination percentage (GP), and time to 50% of germination (*t*_50_, days) of fresh seeds and viability loss index (VLI) for each treatment, species, and year of collection. Data are the mean (standard error) and are used for comparisons. Values with different lowercase letters indicate significant differences between years for each treatment, and values with different uppercase letters indicate significant differences between treatments for each year (DGC test, *p* < 0.05). The dash means that no data are available.

		*C. balansae*			*C. fissilis*		
Treatment		2013	2015	2016	2013	2014	2016
Control (Fresh)	Seed mass	23.09 (0.51) ^b^	26.48 (0.25) ^a^	27.21 (0.39) ^a^	20.34 (0.30) ^b^	25.69 (0.68) ^a^	26.42 (1.72) ^a^
	Lipid content	13.54 (0.29) ^a^	14.21 (0.22) ^a^	13.87 (0.13) ^a^	16.24 (0.01) ^b^	19.21 (0.42) ^a^	16.72 (0.62) ^b^
	Viability	80	100	100	100	100	98
	MC	5.51 (0.11) ^bB^	6.85 (0.10) ^aC^	6.87 (0.08) ^aD^	6.29 (0.05) ^bB^	7.46 (0.25) ^aD^	6.99 (0.10) ^aC^
	GP	59 (6) ^bA^	95 (2) ^aA^	99 (1) ^aA^	98 (2) ^aA^	95 (3) ^aA^	98 (2) ^aA^
	*t*_50_	7.08 (0.41) ^aA^	7.83 (0.35) ^aB^	7.22 (0.30) ^aB^	7.54 (0.13) ^aA^	7.30 (0.43) ^aB^	8.50 (0.42) ^aB^
LiCl	MC	3.85 (0.11) ^bC^	3.81 (0.02) ^bD^	4.94 (0.22) ^aE^	4.12 (0.07) ^aC^	4.25 (0.07) ^aE^	4.29 (0.01) ^aD^
(13%RH, 22 °C)	GP	59 (5) ^bA^	93 (5) ^aA^	94 (3) ^aA^	89 (1) ^aB^	82 (3) ^aB^	84 (9) ^aB^
	*t*_50_	7.73 (0.21) ^aA^	7.88 (0.02) ^aB^	7.77 (0.22) ^aB^	7.82 (0.32) ^aA^	6.45 (0.23) ^bB^	8.71 (0.54) ^aB^
	VLI	−0.04 (0.17) ^aA^	0.02 (0.05) ^aB^	0.05 (0.03) ^aB^	0.09 (0.01) ^aA^	0.13 (0.02) ^aB^	0.14 (0.03) ^aB^
CaNO_3_	MC	6.00 (0.08) ^cA^	6.51 (0.07) ^bC^	8.34 (0.04) ^aC^	6.67 (0.09) ^bA^	8.89 (0.27) ^aC^	6.94 (0.18) ^bC^
(50%RH, 22 °C)	GP	61 (3) ^bA^	92 (2) ^aA^	91 (3) ^aA^	89 (5) ^aB^	80 (2) ^aB^	84 (4) ^aB^
	*t*_50_	7.46 (1.20) ^aA^	7.99 (0.35) ^aB^	7.40 (0.36) ^aB^	7.60 (0.26) ^bA^	6.56 (0.12) ^bB^	8.66 (0.50) ^aB^
	VLI	−0.08 (0.17) ^aA^	0.03 (0.03) ^aB^	0.08 (0.03) ^aB^	0.09 (0.06) ^aA^	0.15 (0.03) ^aB^	0.14 (0.03) ^aB^
NaCl	MC	-	11.55 (0.21) ^bB^	13.11 (0.06) ^aB^	-	13.57 (0.27) ^aB^	13.11 (0.04) ^aB^
(75%RH, 22 °C)	GP	-	87 (4) ^aA^	91 (3) ^aA^	-	76 (3) ^aB^	73 (4) ^aB^
	*t*_50_	-	8.26 (0.15) ^aB^	7.95 (0.14) ^aB^	-	7.48 (0.13) ^bB^	10.16 (0.25) ^aA^
	VLI	-	0.08 (0.05) ^aB^	0.08 (0.02) ^aB^	-	0.19 (0.06) ^aB^	0.26 (0.03) ^aA^
KNO_3_	MC	-	18.78 (0.06) ^bA^	23.96 (0.55) ^aA^	-	23.66 (0.64) ^aA^	25.49 (0.23) ^aA^
(90%RH, 22 °C)	GP	-	64 (4) ^aB^	62 (7) ^aB^	-	64 (4) ^aC^	61 (4) ^aC^
	*t*_50_	-	11.21 (0.19) ^aA^	10.20 (0.95) ^aA^	-	9.67 (0.61) ^bA^	11.36 (0.13) ^aA^
	VLI	-	0.33 (0.04) ^aA^	0.37 (0.07) ^aA^	-	0.32 (0.03) ^aA^	0.38 (0.04) ^aA^

**Table 2:** Estimates of seed longevity parameters (*K_i_*: initial seed viability, *σ*: time for viability to fall by 1 normal equivalent deviates (NED), *σ*^−1^: slope of survival curve, and *p*_50_: time to fall 50% viability) during storage at 35 °C and different equilibrium relative humidity (eRH) and moisture content (MC) for each species and year of collection. Values with different lowercase letters indicate significant differences between years and values with different uppercase letters indicate significant differences between treatments (DGC test, *p* < 0.05).

Species	Year	eRH (%)	MC (%)	*K_i_* (NED)	*σ* (d)	*σ*^−1^ (d^−1^)	*p*_50_ (d)
*Cedrela balansae*	2013	60	9.0	1.26 (0.004) ^b^	24.36 (0.03) ^a^	−0.0411 (0.0400) ^b^	30.93 (0.03) ^b^
	2015	45	5.2	2.08 (0.11) ^A^	97.87(3.27) ^A^	−0.0103 (0.0003) ^D^	202.87 (5.49) ^A^
	2015	60	9.0	2.26 (0.12) ^aA^	18.42 (0.98) ^bB^	−0.0548 (0.0029) ^aC^	41.45 (1.57) ^aB^
	2015	75	11.2	2.23 (0.18) ^A^	5.79 (0.40) ^C^	−0.1752 (0.0117) ^B^	12.75 (0.56) ^C^
	2015	90	18.8	2.34 (0.11) ^A^	4.46 (0.11) ^C^	−0.2247 (0.0056) ^A^	10.39 (0.33) ^C^
*Cedrela fissilis*	2013	60	8.9	1.50 (0.17) ^a^	19.20 (0.37) ^b^	−0.0521 (0.0100) ^a^	28.85 (3.74) ^b^
	2014	45	6.0	1.61 (0.05) ^A^	98.89 (4.32) ^A^	−0.0102 (0.0005) ^D^	158.25 (3.78) ^A^
	2014	60	8.9	1.80 (0.06) ^aA^	26.36 (1.59) ^aB^	−0.0384 (0.0023) ^bC^	47.19 (2.60) ^aB^
	2014	76	12.4	1.70 (0.10) ^A^	11.36 (0.63) ^C^	−0.0889 (0.0046) ^B^	19.16 (0.60) ^C^
	2014	89	22.9	1.53 (0.02) ^A^	8.73 (0.40) ^C^	−0.1153 (0.0053) ^A^	13.29 (0.50) ^C^

**Table 3:** Estimate of *p*_50_ and *σ* of seeds of *Cedrela balansae* and *C. fissilis* using the seed viability equation with estimated constants derived of the model. For both species, seeds were assumed to have an initial viability of 95% (1.64 NED). Three seed moisture contents, including at time of dispersal (6.8%), in equilibrium with 15% relative humidity (RH) at 15 °C (3–5%MC; seed bank storage), and in equilibrium with 50% RH at 22 °C (8–9%MC; ambient room conditions), and three storage temperatures, i.e., −20 °C and 5 °C (seed bank storages) and 22 °C (ambient room temperature), were modelled. Values represent estimated *σ* and *p*_50_ in days and (years). Estimates were calculated using the viability tools in the Seed Information Database [41].

Species	MC (%)	Storage Temperature (°C)					
		−20		5		22	
		*p*_50_	*σ*	*p*_50_	*σ*	*p*_50_	*σ*
*Cedrela balansae*	6.8	9813.7 (26.9)	5705.2 (15.6)	2388.1 (6.5)	1388.3(3.8)	397.5 (1.1)	231.1
	3.8	41,996.6 (115.1)	24,414.9 (66.9)	10,219.7 (28.0)	5941.2 (16.3)	1701.1 (4.7)	989.0 (2.7)
	8.3	5964.2 (16.3)	3467.3 (9.5)	1451.4 (4.0)	843.8 (2.3)	241.6	140.4
*Cedrela fissilis*	6.8	1165.6 (3.2)	677.6 (1.9)	283.6	164.9	47.2	27.4
	4.3	2624.0 (7.2)	1525.5 (4.2)	638.5 (1.7)	371.2 (1.0)	106.3	61.8
	8.9	723.8 (2.0)	420.8 (1.2)	176.1	102.4	29.3	17.0

**Table 4:** Spearman’s rank correlation probabilities of seed mass (SM), moisture content (MC), lipid content (LC), viability loss index (VLI), initial probit seed viability (*K_i_*), standard deviation (*σ*), time for viability to fall 50% viability (*p*_50_), annual climatic variables (maximum: MaAT, mean: MeAT, and minimum: MiAT annual temperatures), accumulated precipitation (APP) and climatic variables during fruiting period (maximum MaT_J-J_, mean: MeT_J-J_, and minimum temperatures: MiT_J-J_), and accumulated precipitation (APP_J-J_) for both species. Probabilities in bold are significant (*p* < 0.05).

	*Cedrela balansae*							*Cedrela fissilis*						
	SM	MC	LC	VLI	*K_i_*	*σ*	*p*_50_	SM	MC	LC	VLI	*K_i_*	*σ*	*p*_50_
MC	**0.007**							**0.018**						
LC	0.448	0.130						0.054	0.060					
VLI	0.466	0.839	0.483					0.438	0.917	0.523				
*K_i_*	**0.003**	**0.013**	0.151	0.955				0.225	0.277	0.110	0.827			
*σ*	**0.016**	**0.030**	0.106	0.774	**0.001**			0.482	0.338	0.655	0.959	0.338		
*p*_50_	**0.035**	**0.035**	0.058	0.608	**0.003**	**0.010**		0.085	0.142	0.277	0.658	0.064	0.991	
MaAT	**0.015**	**0.009**	0.350	0.782	**0.004**	**0.003**	**0.004**	**0.031**	0.120	0.053	0.890	**0.042**	0.991	**0.042**
MeAT	**0.015**	**0.009**	0.350	0.782	**0.003**	**0.004**	**0.004**	**0.031**	0.120	0.053	0.890	**0.042**	0.090	**0.042**
MiAT	0.855	0.901	0.217	0.678	**0.005**	**0.004**	**0.004**	**0.030**	0.119	0.054	0.895	**0.040**	0.991	**0.041**
APP	**0.015**	**0.009**	0.350	0.782	**0.005**	**0.003**	**0.003**	0.581	0.119	0.459	0.782	**0.042**	0.090	**0.041**
MaT_J-J_	**0.006**	**0.010**	0.058	0.890	**0.003**	**0.003**	**0.004**	0.581	0.120	0.459	0.782	**0.042**	0.092	**0.040**
MeT_J-J_	**0.015**	**0.009**	0.350	0.782	**0.004**	**0.004**	**0.004**	0.581	0.120	0.460	0.785	**0.040**	0.991	**0.042**
MiT_J-J_	**0.015**	**0.010**	0.350	0.782	**0.004**	**0.003**	**0.004**	0.581	0.119	0.460	0.782	**0.040**	0.090	**0.041**
PP_J-J_	**0.006**	**0.009**	0.059	0.890	**0.003**	**0.003**	**0.003**	0.581	0.120	0.459	0.782	**0.042**	0.991	**0.042**

## Data Availability

Due to privacy issues, the data presented in this study are available on request from the corresponding author.

## Supplementary Materials

The following supporting information can be downloaded at: https://www.mdpi.com/article/10.3390/plants15030380/s1, Figure S1: Annual climate trends in study sites.
