# Supplementary figures and images for: Interannual Variation in Seed Traits of Cedrela Species: Implications for Conservation in the Context of Climate Change
Source: Plants (Basel). 2026 Jan 26;15(3):380. doi: 10.3390/plants15030380 (PMC12899300; doi:10.3390/plants15030380)

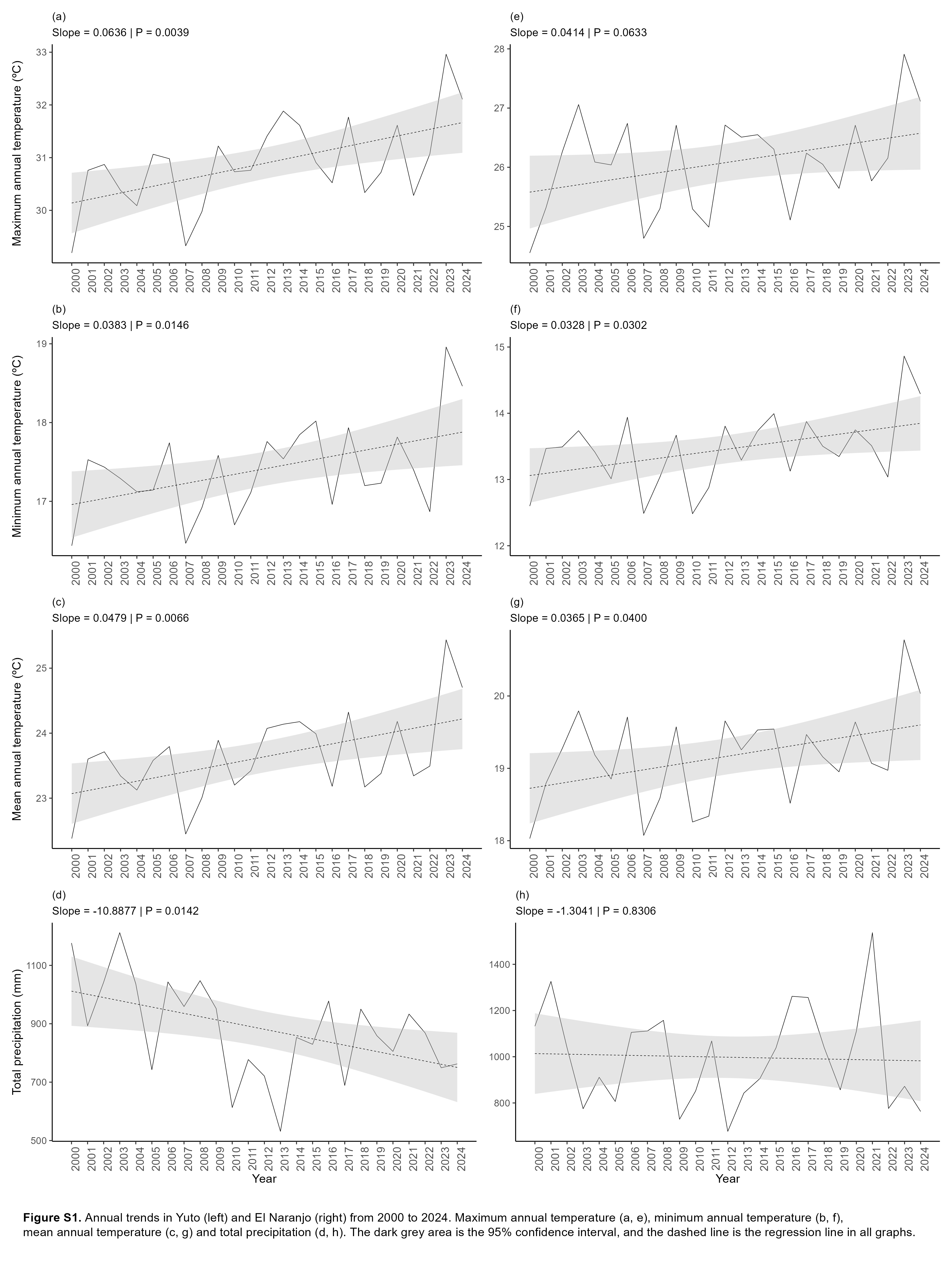

Supplement: Supplementary file 1 [file plants-15-00380-s001.zip › plants-4101600-supplementary.png]
